# A procedure to quantify the feed intake response of growing pigs to perturbations

**DOI:** 10.1017/S1751731119001976

**Published:** 2019-08-23

**Authors:** H. Nguyen-Ba, J. van Milgen, M. Taghipoor

**Affiliations:** 1 PEGASE, Agrocampus Ouest, INRA, 35590 Saint-Gilles, France; 2 Faculty of Animal Science, Vietnam National University of Agriculture, Hanoi, Vietnam; 3 MoSAR, AgroParisTech, INRA, Université Paris-Saclay, 75005 Paris, France

**Keywords:** modelling, resistance, resilience, health, breeding

## Abstract

Improving robustness of farm animals is one of the goals in breeding programmes. However, robustness is a complex trait and not measurable directly. The objective of this study was to quantify and characterize (elements of) robustness in growing pigs. Robustness can be analysed by examining the animal’s response to perturbations. Although the origin of perturbations may not be known, their effect on animal performance can be observed, for example, through changes in voluntary feed intake. A generic model and data analysis procedure was developed (1) to estimate the target trajectory of feed intake, which is the amount of feed that a pig desires to eat when it is not facing any perturbations; (2) to detect potential perturbations, which are deviations of feed intake from the estimated target trajectory; and (3) to characterize and quantify the response of the growing pigs to the perturbations using voluntary feed intake as response criterion. The response of a pig to a perturbation is characterized by four parameters. The start and end times of the perturbation are ‘imposed’ by the perturbing factor, while two other parameters describe the resistance and resilience potential of the pig. One of these describes the immediate reduction in daily feed intake at the start of the perturbation (i.e., a ‘resistance’ trait) while another parameter describes the capacity of the pig to adapt to the perturbation through compensatory feed intake to rejoin the target trajectory of feed intake (i.e., a ‘resilience’ trait). The procedure has been employed successfully to identify the target trajectory of feed intake in growing pigs and to quantify the pig’s response to a perturbation.

## Implications

The study provides a data analysis procedure to detect the impact of perturbations on feed intake in growing pigs, and a mathematical model to quantify traits related to resistance and resilience. When pigs are kept in the same environment and are facing a common perturbing factor, the model can be used to identify differences in resistance and resilience among pigs, which can be used in selection programmes. Although this procedure uses feed intake as a response criterion and is applied to growing pigs, it is generic and can be applied to other species and with other response criteria.

## Introduction

Growing pigs, like other animals, are confronted with variation in their environment to which they may have to respond. This includes the effects of climate change (e.g., periods of extreme weather), infectious diseases, but also management practices and interactions with other animals. Robustness deals with the way animals respond to changes in their environment. Knap (2005) defined robust pigs as ‘pigs that combine high production potential with resilience to external stressors, allowing for unproblematic expression of high production potential in a wide variety of environmental conditions’. Robustness is a complex concept, which is difficult to quantify and characterize because it includes multiple dynamic elements such as the rates of response to and recovery from environmental perturbations (Friggens *et al*., 2017). The response of an animal to a perturbation can be described in terms of resistance and resilience, which are defined as the capacity of an animal to minimize impacts of perturbing factors and to quickly return to the pre-perturbed condition (De Goede *et al*., 2013; Colditz and Hine, 2016).

Because of the dynamic nature of the response of animals to a perturbation, it is difficult to use single time-point measurements to quantify resistance and resilience (Friggens *et al*., 2017). Recent developments in monitoring technologies allow the continuous recording of animal performance (Neethirajan, 2017). Although several studies have explored these technologies to study the impacts of perturbations on animal performance (Codrea *et al*., 2011; Munsterhjelm *et al*., 2015; Friggens *et al*., 2016), these technologies have not yet been used to develop a generic method that detects perturbations and that allows to quantify the animal’s response to perturbations. Therefore, the objective of this study was to propose a data analysis and modelling procedure to detect the impact of perturbations in growing pigs and quantify the feed intake response in terms of resistance and resilience.

## Material and methods

### General description of the model

Perturbations such as heat stress or sanitary challenges typically have a transitory impact on the pig, resulting in changes in feed intake and BW gain. Although the cause of a perturbation is not always known, the consequences on animal performance can be observed. Because of the rapid development in monitoring technologies on farm, feed intake can now be recorded in individual pigs with a very high frequency (i.e., up to the level of meal intake patterns). Moreover, feed intake is among the first measurable and non-invasive traits affected by perturbations, and was therefore considered as a suitable trait to quantify the response of a pig to a perturbation.

Only perturbations that have a negative impact on feed intake are considered in this study. Perturbations that result in an increase in feed intake (e.g., cold stress, immuno-castration or providing a diet with low-energy content) are not considered here, but the proposed method is generic and can be adapted to account for these types of perturbations.

It is hypothesized that the observed cumulative feed intake (**CFI**) of a pig is the combination of a target trajectory curve (i.e., the amount of feed a pig desires to consume in a non-perturbed condition) and a change in feed intake due to perturbations. During a perturbation, the feed intake of the pig will deviate from the target trajectory but, once the perturbing factor is over, the pig will strive to increase its feed intake through compensatory feed intake to rejoin the target trajectory of CFI (**target CFI**).

The data analysis procedure includes two main steps. The first one is the estimation of the target trajectory curve of feed intake. Deviations of the observed feed intake from this target trajectory represent the potential consequence of a perturbation, and a classification process is performed to identify the most important deviations. The second step is the quantification of response of the animal in terms of resistance and resilience. In short, the procedure is based on two model components: one estimates the target trajectory of feed intake and the other one characterizes the perturbation.

Although daily feed intake (**DFI**) is often used as a production trait, fluctuations in DFI data make detection of perturbations difficult. Moreover, after a perturbed period, the overall reduction in feed intake needs to be compensated for by an equal increase in feed intake during the recovery period, which should surpass the target trajectory of DFI (**target DFI**). The cumulative feed intake (i.e., the integral of DFI) has the advantage over DFI of being less variable and, more importantly, allows for an easier representation of a trajectory including deviations and recovery. In the absence of a perturbation, it is hypothesized that the observed CFI is identical to the target CFI. During a perturbation, the observed CFI deviates from the target CFI (i.e., it increases to a lesser extent) and, once the perturbing factor is over, the animal seeks to rejoin the target CFI through compensatory feed intake, without surpassing it in a systematic way.

### Estimation of the target trajectory of feed intake and detection of perturbations

The target trajectory of CFI is the amount of feed a pig desires to eat when it is in a non-perturbed condition. The target trajectory of CFI was described by an empirical polynomial function of time, without pretending a mechanistic cause. The reason for this is that feed intake was recorded on a daily basis and is statistically the independent variable. Preliminary analyses indicated that using a third-order polynomial of CFI in combination with a perturbation model could result in biologically unrealistic predictions for DFI. We therefore defined the model of target DFI so that it can either increase with time or remain constant, resulting in the so-called linear-plateau model for DFI. Consequently, the target CFI was described by a quadratic-linear function of time:
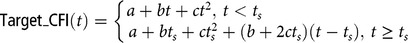
(1)


where ‘*t*’ is the age of the animal (days) and ‘Target_CFI(*t*)’is the target CFI at day ‘*t*’. The parameters *a*, *b* and *c* are the classical parameters of a polynomial function, and *t*_*s*_ is the day when the quadratic segment of the curve changes to the linear segment.

To facilitate the biological interpretation of the parameters, equation (1) was reparametrized by replacing parameters *a*, *b* and *c* by *t*_0_ (the estimated age at which CFI = 0), CFI_mid-point_ (the estimated CFI at the mid-point determined halfway between *t*_0_ and the last observation) and CFI_last_ (the estimated CFI at the last observation). Details of this re-parametrization are described in Supplementary Material S1.

Using the reparametrized equation (1) to estimate the target CFI, two possible problems were encountered with the resulting linear-plateau function for DFI. Firstly, the linear segment can have a very modest negative slope, which would mean that the DFI decreased slightly with increasing age. In that case, a constant value for DFI was assumed (rather than a linear-plateau model), resulting in a linearly increasing function for CFI (with two parameters *t*_0_ and CFI_last_). Secondly, to avoid the estimation of *t*_s_ being too close to either the first or the last observation, a linear function was then used to describe DFI, resulting in a quadratic function for CFI (with three parameters *t*_0_, CFI_mid-point_ and CFI_last_).

To estimate the target CFI, the reparametrized equation (1) has to be fitted to non-perturbed data. Therefore, a statistical procedure was used to successively eliminate observations that could result from perturbed periods. Observations that are consistently below the fitted curve may correspond to feed intake during perturbed periods. An auto-correlation test was used as a selection criterion to temporarily remove data with negative residuals from the dataset. The fitting procedure was then repeated on the resulting dataset until the auto-correlation of the residuals was no longer significant. Compared to fitting the curve to the original dataset of CFI, this procedure results in moving the CFI curve upwards, while fitting the model to fewer observations compared to the original dataset. Preliminary analysis indicated that the absence of auto-correlation could be achieved only if very few data remained. This appears to be due to small oscillations in CFI that are not necessarily the result of perturbations. To ensure that the target CFI is estimated with a reasonable number of observations, the data elimination procedure was terminated when at least 20 observations were remaining. In short, the parameters of the target CFI were estimated by repeatedly fitting the reparametrized equation (1) to CFI data and temporarily eliminating observations with negative residuals until there was no auto-correlation among the residuals or when at least 20 observations remained.

Deviations from the target CFI correspond to potential perturbations. As indicated earlier, small oscillations in feed intake patterns exist. The aim here is to detect the most important deviations that are the result of perturbations. These perturbations can then be characterized by the duration and magnitude of the deviation from the target CFI. A deviation was considered as a perturbation if it lasted at least 5 days, to ensure that a reasonable number of data were used to estimate the model parameters. The magnitude was determined by calculating the maximum reduction of a deviation from the target trajectory. Because CFI is increasing continuously, deviations from the target CFI were expressed as a percentage and an arbitrary value of 5% was set as a threshold value to identify a perturbation. To identify perturbations among all the deviations from the target CFI, a B-spline function of order 6 was fitted to the difference between the observed CFI and the target CFI. Any period during which the observed data deviated from the target CFI for more than 5 days and for more than 5% was considered to be the result of a perturbation. The interest of using a B-spline function is its high flexibility and its smoothing properties that allow to capture small deviations from the target CFI (Ramsay and Silverman, 2007).

Deviations that occurred only during the first week were not considered because pigs may encounter many stressors during this period (e.g., mixing of groups) and deviations from the target CFI of more than 5% occur frequently due to the small value of CFI during the first week. However, deviations that started during the first week and for which the selection criteria of duration and magnitude continue to hold during the second week were considered the result of a perturbation.

### Characterization of the response to a perturbation

**Figure 1:**
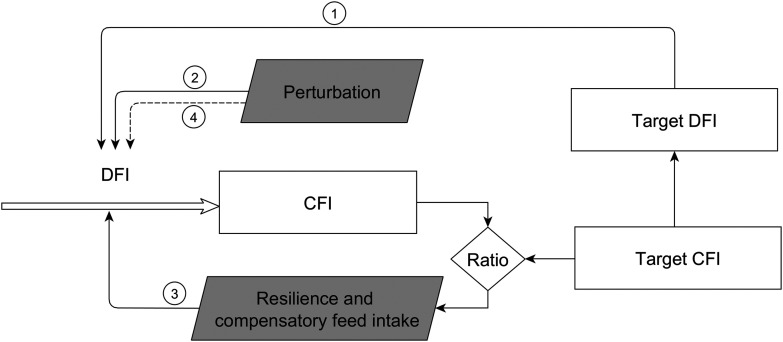
General mechanism of a model that quantifies the pig’s response to a perturbation. Solid arrows indicate causal relationships in the model, the double arrow indicates the flux and the dashed arrow indicates the disappearance of perturbing factor. Numbers indicate the response elements: (1) in the absence of a perturbation, the daily feed intake (DFI) is equal to the target DFI; (2) the initiation of a perturbation has a negative and constant effect on DFI and, because of the reduction in DFI, the cumulative feed intake (CFI) starts to deviate from the target CFI; (3) the ratio between the CFI and the target CFI triggers the pig’s resilience mechanism to limit the effect of the perturbation on DFI; (4) once the perturbing factor is over, its negative effect on DFI disappears, but the resilience mechanism is still active resulting in compensatory feed intake allowing the CFI to approach the target CFI.

**Figure 2:**
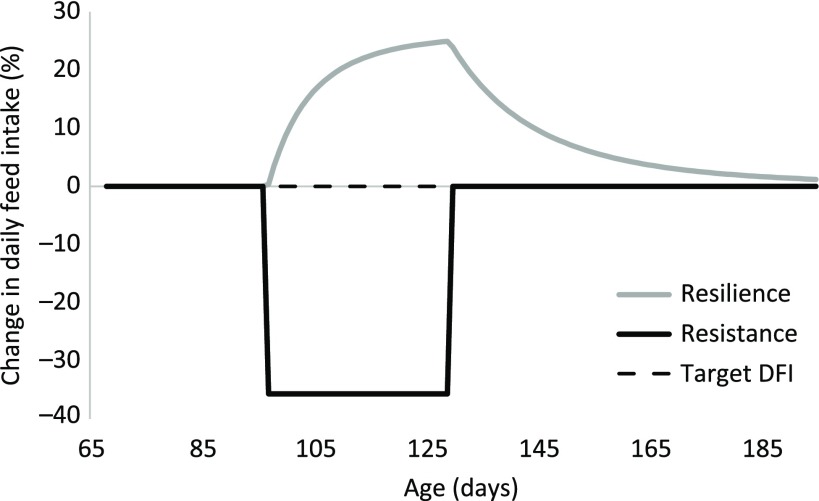
Mechanisms that determine the response of a pig to a perturbation. The perturbation is estimated to occur between around days 97 and 129 of age. The dashed line indicates the target trajectory of daily feed intake (DFI). The black line indicates the constant and negative impact of the perturbation on the pig (resistance), resulting in a 35.6% reduction in DFI. The grey line represents the resilience capacity (during the perturbation) and compensatory feeding of the pig (after day 129).

The model to characterize the animal’s response to a perturbation is based on an ordinary differential equation and includes two components: the immediate impact of the perturbation and the response of the pig to the perturbation (Figure 1). A perturbation was assumed to have an instantaneous, negative and constant impact on the DFI of the pig for the duration of the perturbing factor. The reduction in DFI will result in that the CFI deviates progressively from the target CFI. The ratio between CFI and the target CFI is used as a driving force to trigger the pig’s resilience mechanism in a proportional way (Figure 1). The smaller the ratio between the CFI and the target CFI, the greater will be the intensity of resilience mechanism for DFI. The change in DFI due to perturbation (compared with the target DFI) is the sum of the components depicted by resistance (Figure 2, black line) and resilience including compensatory feed intake (Figure 2, grey line). During the perturbed period, the resilience mechanism limits the effect of the perturbing factor. As indicated in Figure 2, at the end of the perturbed period, the negative effect of the perturbation (around −35%) is partially compensated for by the resilience mechanism (around + 25%). Once the perturbing factor is over, the negative effect on DFI disappears, but the CFI ratio will still be smaller than one. This results in compensatory DFI where the observed DFI will be greater than the target DFI that, in turn, results in that the CFI will approach the target CFI.

The perturbation model was conceptualized in a way that the impact of a perturbation on feed intake can be characterized by four parameters. Two parameters indicate the start (t_start) and end times (t_stop) of the perturbing factor, while the third parameter (*k*1) describes the constant negative impact of the perturbation on DFI. The fourth parameter (*k*2) is the marginal response in DFI due to a change in the ratio between the CFI and the target CFI, and describes the capacity of the pig to adapt to the perturbation through resilience and compensatory feed intake. The perturbation model is therefore the result of resistance and resilience mechanisms and can be written as:
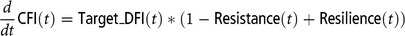
(2a)where ‘Resistance(*t*)’ takes the value of *k*1 between the t_start and t_stop time, and is zero otherwise, while ‘Resilience(*t*)’is described by:
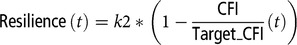
(2b)


It is acknowledged that the proposed procedure includes a number of arbitrary elements. This concerns the choice of feed intake as the only response criterion, the model choices for the target trajectory for CFI and for the DFI during and after the perturbed periods, and the step-wise method to quantify the animal’s response. However, the method is generic in that the different elements can be changed and adapted as judged necessary.

### Data source used for model calibration

Data were collected from an experimental farm of INRA in Le Magneraud (Charente-Maritime, France). Five pigs from the same batch (i.e., they were born on the same farm and approximately on the same day) were chosen to demonstrate the procedure. The pigs entered the same growing facility at 68 days of age and stayed there until reaching their slaughter weights (124 kg on average). Feed was provided *ad libitum*. Feed intake was recorded on a daily basis using the single-place Acema 64 electronic feeder (Acemo, Pontivy, France) as described by Labroue *et al*. (1994). Because CFI is sensitive to missing data (e.g., due to loss of a radio-frequency identification (**RFID**) ear tag or malfunctioning of the feeder), a procedure to deal with missing data was developed (Supplementary Material S2; an example of missing data estimation by the procedure is shown in Figure S1). There were no missing feed intake records for the five pigs used here to illustrate the procedure. Because pigs were fasted one day before leaving to slaughterhouse, the last observation of feed intake of each pig was ignored.

### Statistical analysis

All statistical and optimization procedures were performed using R software version 3.5.0 (http://cran.r-project.org/). To account for scale differences in the target CFI (reparametrized equation (1)), a weighted regression procedure was applied using (1/CFI)^2^ as statistical weight. The optimization was performed using the non-linear function ‘nlsLM’ of the package ‘minpack.lm’. The structural identifiability of reparametrized equation (1) and equations (2a) and (2b) was tested using the software DAISY (Bellu *et al*., 2007). All equations were structurally identifiable, meaning that the parameter estimation problem is well posed and it is theoretically possible to estimate uniquely the model parameters given the available measurements (Muñoz-Tamayo *et al*., 2018).

The test for auto-correlation was performed by a Wald-Wolfowitz runs test. To fit the B-spline function to the difference between the observed CFI and target CFI, the package ‘fda’ was used. To characterize the pig’s response to a perturbation, equations (2a) and (2b) were solved using the ‘ode’ function of the ‘desolve’ package with an integration step size (dt) of one day. The optimization was done using the ‘optim’ function.

## Results

The procedure is illustrated step by step for one of the pigs, and the results for all five pigs are presented in Table 1.

**Table 1:** Parameter estimates of the target trajectory of cumulative feed intake (CFI) and of the perturbation model to characterize the response to a perturbation of five grouped-housed growing pigs

Pig				Parameter estimates of the target CFI model				Parameter estimates of the perturbation model			
	Function type^1^	Remaining data^2^	*t*_last_ (day)	*t*_0_ (day)	*t*_*s*_ (day)	CFI_mid-point_ (kg)	CFI_last_ (kg)	t_start (day)	t_stop (day)	*k*1 (%)	*k*2
01	QL	37	195	67.3	162	155	334	96.9	129	−35.6	2.81
02	L	37	195	81.8			231	97.7	134	−68.6	1.26
03	QL	22	214	67.9	133	109	260	96.7	152	−52.6	2.73
04	Q	32	195	67.2		149	326	100	151	−51.5	5.13
05	Q	25	194	66.6		157	331	103	154	−49.1	5.13

*t*_last_ = the last observed age in the growing period; *t*_0_ = estimated age at which CFI = 0; *t*_*S*_ = the age when the quadratic segment changes to the linear segment in the quadratic-linear function; CFI_mid-point_ = estimated CFI at the mid-point of the growing period; CFI_last_ = estimated CFI at t_last_; t_start = the day the perturbing factor started; t_stop = the day the perturbing factor ended; *k*1 = instantaneous reduction in daily feed intake at t_start; *k*2 = resilience parameter.

1 Function type for the target CFI: QL = quadratic-linear function; Q = quadratic function; L = linear function.

2 Remaining data: number of observations used to estimate the target CFI.

**Figure 3:**
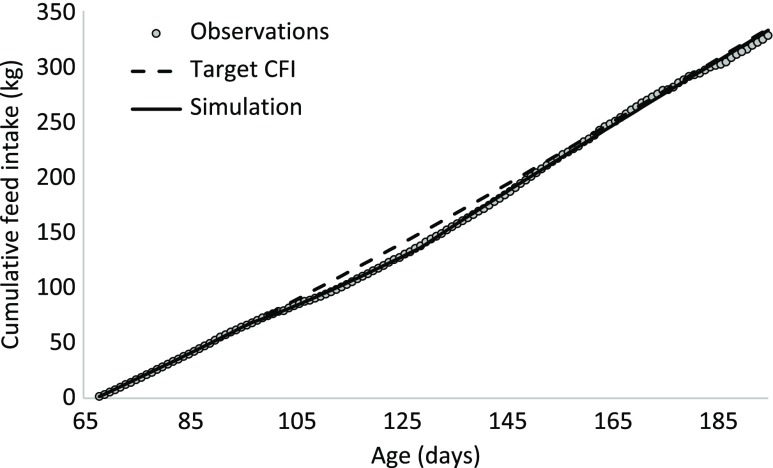
Cumulative feed intake (CFI) of a pig in response to a perturbation. The target CFI is described by a quadratic-linear model (the change in model segments occurred at 162 days). The perturbation was estimated to occur between 97 and 129 days of age, resulting in a deviation of the CFI from the target CFI.

The target trajectory of CFI of pig 01 was estimated using the quadratic-linear function of the reparametrized equation (1). The auto-correlation test was conducted to temporarily remove observations associated with perturbed periods. The process was terminated when 37 CFI observations remained, even though auto-correlation still existed. Further application of the procedure would result in fewer than 20 remaining observations. Estimated parameters of the target CFI are indicated in Table 1. Figure 3 shows the target CFI against observed CFI, illustrating that the observed CFI deviated from the target CFI from 100 to 150 days of age and, to a lesser extent after 185 days of age.

**Figure 4:**
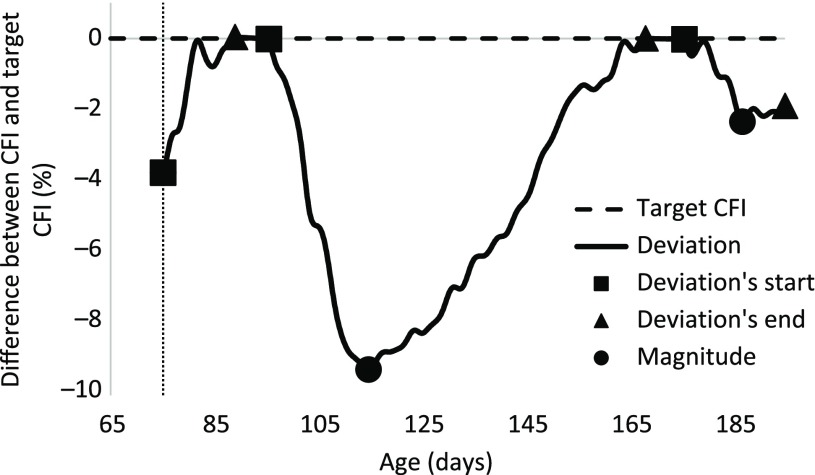
Difference between the observed cumulative feed intake (CFI) and the target CFI of a pig. Three deviations were detected but, based on the selection criteria for a perturbation, only one deviation was considered as a perturbation. Deviations during the first week of growing period (indicated by the vertical dashed line) were not considered as perturbations.

Figure 4 illustrates the differences between the observed and target CFI. Not considering the data of the first week, three deviations from the target CFI were detected. Given the selection criteria for a perturbation, only one deviation was considered as a perturbation. The analysis using the B-spline function indicated that it lasted from 96 to 168 days of age and the maximum deviation was 9.3%, which occurred at 115 days of age.

**Figure 5:**
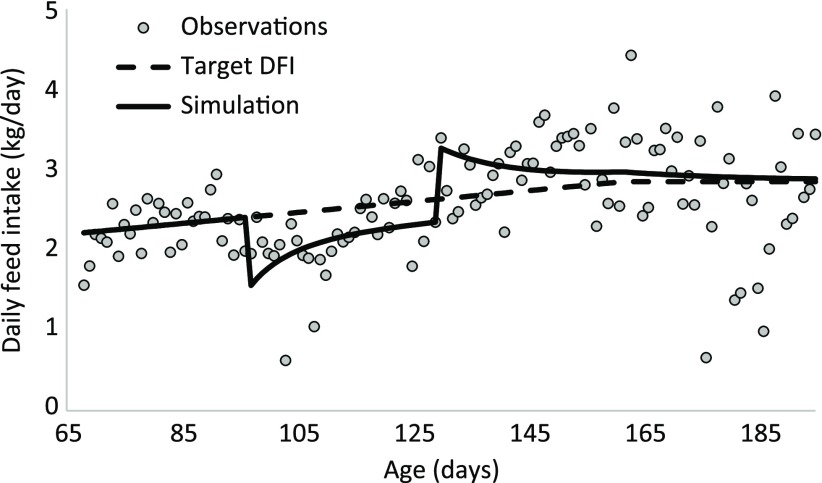
Daily feed intake (DFI) of a pig and modelling results. The perturbing factor induced an immediate reduction in DFI (compared with the target DFI) at the beginning of the perturbed period, which was counteracted by resilience mechanisms of the pig. Once the perturbing factor ended, the pig consumed more feed than the target DFI through compensatory feed intake to recover.

Equations (2a) and (2b) were used to estimate the parameters describing the response of the animal to a perturbation. The period during which the perturbation occurred was estimated to start at 97 days of age (t_start) and to end at 129 days of age (t_stop). The instantaneous reduction in DFI at the onset of the perturbation *k*1 was estimated at 35.6%. The estimated value of the resilience parameter *k*2 was 2.81, which indicates that if the CFI is 1% below the target CFI, the pig would strive to eat 2.81% more compared to its target DFI. At 129 days of age, the negative effect of the perturbing factor stopped, but the resilience mechanism remained active because the CFI was still lower than the target CFI. The ratio between the two was therefore still below one, resulting in compensatory feed intake. The response of this animal to a perturbation is given in Figure 5 for the change in DFI and in Figure 3 for CFI. The maximum deviation in CFI occurred when the perturbation stopped at 129 days. At 130 days, the CFI was 139 kg, which was 9% below the target CFI of 152 kg. This triggered a compensatory feed intake in which the DFI was 24% greater than the target DFI (Figure 5). Because of the compensatory feed intake, the CFI gradually approached the target CFI (Figure 3).

Table 1 shows the estimated parameters of the target CFI and of the response to perturbations of five pigs. The process to temporarily remove observations with negative residuals was always stopped while there was still auto-correlation to ensure retaining at least 20 observations. The procedure indicated that all five pig were affected by one major perturbation during the growing period. Moreover, the start (t_start) and the last (t_stop) days of the estimated perturbation were approximately similar for all five pigs (99 ± 2.7 days and 144 ± 11.6 days, respectively). However, values of the response traits (*k*1 and *k*2) varied between these pigs (from −68.6% to −35.6% for *k*1; and from 1.26 to 5.13 for *k*2).

## Discussion

Characterization and quantification of the response of animals to perturbations are important in animal management and breeding. The recent and rapid development of monitoring devices in combination with data analysis and modelling techniques offer a great potential to progress in this area. This study illustrated how DFI records can be used to characterize and quantify elements of resistance and resilience in growing pigs.

### Difficulties in modelling the response of animals to perturbations

Attempts to quantify the animal’s response to perturbations have been made both statistically and conceptually. However, traditional single time-point recording of performance traits cannot capture the whole process in which the response to and recovery from perturbations of an animal occurs (Friggens *et al*., 2017). For example, Pastorelli *et al*. (2012) conducted a meta-analysis to study the consequence of six sanitary perturbations on feed intake and growth in growing pigs. However, only a small number of experiments in the literature were available that allowed to quantify the response of the animals to these perturbations. They differentiated the response of animals to the different perturbations but, due to the type of data reported in the literature, they could only report the response as an average reduction in feed intake and/or daily gain for the whole experimental period. Mechanistic models that represent the dynamic response of animals to a perturbation have been developed based on conceptual grounds (Wellock *et al*., 2003b; Sandberg *et al*., 2006). However, despite their theoretical interest, there has been little high-frequency data thus far to challenge the proposed concepts and to ensure practical application of these models. These kinds of data are required to detect, understand and quantify the response of an animal to a perturbation (Codrea *et al*., 2011; Wallenbeck and Keeling, 2013; Munsterhjelm *et al*., 2015).

### Modelling the target trajectory of feed intake

In modelling growth and feed intake, different approaches towards ‘cause’ and ‘effect’ have been used. In the ‘push approach’, feed intake is often described as a simple function of time or BW driving growth. Frequently used functions include the monomolecular function (i.e., an exponentially declining function with an asymptote), the power function and a Gamma-function describing feed intake relative to maintenance energy requirements (Van Milgen *et al*., 2008; Black, 2009; National Research Council, 2012). In the ‘pull approach’, functions for desired growth (i.e., protein and lipid deposition) are defined which, in combination with aspects of energy metabolism, result in a desired feed intake. Most feed intake models describe DFI, rather than CFI. In an analysis of different growth functions, Schulin-Zeuthen *et al*. (2008) indicated that BW could very well be described by a monomolecular function of CFI, basically indicating that animals grow because they eat and, at maturity, they eat for maintenance.

It is interesting to note that for all five animals used in this study, a significant auto-correlation remained for CFI unless the procedure was allowed to proceed beyond the limit of 20 remaining observations. The presence of auto-correlation in CFI data indicates that there are patterns in feed intake that cannot be captured by a polynomial model with (potentially) four parameters. Although the choice of 20 observations was arbitrary, it is a compromise between the number of remaining data and the presence of auto-correlation in the target CFI. If the filtration procedure was allowed to go further, there would be no data with auto-correlated residuals, but the estimation of the target CFI (which is described by maximum four parameters) would be based on a small number of observations. In contrast, if the procedure was stopped earlier (with more remaining observations), the target CFI would include more data with auto-correlated residuals, some of which could be due to a perturbation.

### Modelling the response to perturbations

In describing the response of an animal to a perturbation, Wellock *et al*. (2003a) and Sandberg *et al*. (2006) used a pull approach to describe the feed intake response. This approach is probably biologically more appropriate than the empirical push approach that was used in this study, but it requires an explicit representation of the nutrient requirements for growth and those related to the perturbation (e.g., for the immune response). Perturbations can have both direct and indirect effects on performance through metabolism and nutrient utilization (Le Bellego *et al*., 2002; Pastorelli *et al*., 2012). However, when feed intake is the only measured response trait, it is difficult to disentangle these direct and indirect effects.

To characterize resistance and recovery capacity of the animal, Sandberg *et al*. (2006) proposed a model of the DFI response to a pathogen challenge. The resistance part of the model in this study (Figure 2, black line) is conceptually similar to the model of Sandberg *et al*. (2006). The difference is that they included a lag time from inoculating the pathogen until the first sign of a reduction in feed intake, which requires knowledge of when the animals are exposed to a pathogen. Also, Sandberg *et al*. (2006) assumed existence of a duration where feed intake gradually decreases to its minimum value, followed by a plateau before it gradually recovered to the reference value. The recovery rate used by that model can be compared to the resilience mechanism proposed in our approach (Figure 2, grey line), which allows for compensatory feed intake to occur. The model of Sandberg *et al*. (2006) does not include an explicit representation of compensatory feed intake, although this may occur through the pull approach used in their model. The existence of compensatory feed intake following perturbations is supported by other studies (Kyriazakis and Emmans, 1992; Pastorelli *et al*., 2012).

The model proposed in this study is also somewhat similar to the spring and damper model developed by Sadoul *et al*. (2015). In that model, which is analogous to a suspension system in a car, the impact of a perturbation is considered as a ‘pulling’ force on the system and resistance and resilience are characterized by the ability of the system to resist from being deformed (i.e., spring) and to reduce the amplitude of the deformation (i.e., damper). After the perturbing force is released, the system recovers by itself and the recovery rate depends on the ratio between the parameter values of the spring and damper. Parameter values of the spring and damper also determine whether oscillations in the response will occur. Although these oscillations may be used to represent compensatory feed intake in terms of DFI, it may be more difficult to use these oscillations to represent compensatory feed intake in terms of CFI.

As illustrated by the models of Sandberg *et al*. (2006), Sadoul *et al*. (2015) and this study, there are different ways to represent the response of animal to a perturbation. At this stage, we aimed to keep the model as generic and as simple as possible, but any aspect of the procedure can be changed as deemed necessary. For example, knowledge about the origin of the perturbation can be helpful in establishing an appropriate perturbation model. In the current model, a specific duration of the perturbing factor is included, which would be appropriate to reflect a period of heat stress. Other perturbing factors such as a viral challenge may have a specific starting point (with or without a lag time), but the duration of the challenge may be less clear. The effect of a viral challenge may be reduced because the perturbing factor becomes less effective by itself or because the animal builds up resilience through its immune response even though the perturbing factor may still be present.

### Possible future developments

The data analysis procedure was applied here to five animals that were raised at the same time in the same environment. The feed intake curves were analysed separately for each pig, but the period of perturbation appeared to be similar for the five pigs (Table 1). It can be speculated that these pigs were challenged by the same perturbing factor, but the responses differed between the five pigs. The proposed data analysis procedure has the potential to be applied on a large number of pigs. For example, it could be used to identify periods during which several pigs (e.g., in the same pen or in the same barn) reduce their feed intake at the same time. If this occurs for a considerable number of pigs in the group, it may be reasonable to assume that all pigs in that group were exposed to the same perturbing factor. This would allow quantifying differences in the responses of individual pigs to a common perturbing factor through the resistance and resilience traits *k*1 and *k*2. Certainly, this has a great potential in animal breeding to estimate heritabilities and to evaluate relationships between performance and robustness traits (Guy *et al*., 2012; Hermesch *et al*., 2015).

Elements of the data analysis procedure proposed in this study can also be used in precision livestock farming. For example, it could be used as an early warning system if deviations in feed intake occur relative to the target CFI. Likewise, specific management strategies (e.g., in terms of nutrition, medication or care) may be given to animals that deviate from their target CFI to limit the impact of the (known or unknown) perturbing factors and to facilitate the recovery of the animals so that they can regain their target CFI.

## Conclusion

The recent development of monitoring technologies offers new opportunities for livestock management. Recording of individual feed intake in group-housed pigs is becoming more accessible, and feed intake can be very informative about the health and welfare status of the animal. The model and data analysis procedure proposed in this study showed to have the potential to detect the impact of a perturbation on the feed intake and to quantify the response of the animal in terms of traits related to resistance and resilience.

## Software and data repository resources

The R code of the procedure described in this article and feed intake data of pig 01 are accessible on the ZENODO data warehouse (DOI: 10.5281/zenodo.3366107). Users need to store all three files in one folder and change the working directory of the file ‘Procedure_quantify_perturbation. Rmd’ accordingly to reproduce the results of this article.

## Supplementary material

For supplementary material accompanying this paper visit https://doi.org/10.1017/S1751731119001976. click here to view supplementary material
