# Supplementary material for: A procedure to quantify the feed intake response of growing pigs to perturbations
Source: Animal. 2019 Aug 23;14(2):253–60. doi: 10.1017/S1751731119001976 (PMC6974428; doi:10.1017/S1751731119001976)
Supplement: Supplementary file 1 [file S1751731119001976sup001.pdf]

*Animal* journal

# A procedure to quantify the feed intake response of growing pigs to perturbations

H. Nguyen-Ba[1, 2, a], J. van Milgen[1], and M. Taghipoor[3]

[1] *PEGASE, Agrocampus Ouest, INRA, 35590 Saint-Gilles, France*

[2] *Faculty of Animal Science, Vietnam National University of Agriculture, Hanoi, Vietnam*

[3] *MoSAR, AgroParisTech, INRA, Université Paris-Saclay, 75005 Paris, France*

[a] *Corresponding author: Hieu Nguyen-Ba. Email: hieu.nguyen-ba@inra.fr*

## S1. An example of function re-parameterization

The function to estimate the target trajectory curve of cumulative feed intake (**CFI**) is described in Equation 1 in the article. Consider the case that the target CFI is represented by a quadratic function then:

$$Target\ CFI(t) = a + bt + ct^2 \qquad \text{(S1)}$$

To reparametrize this model to a new model with three biologically meaningful parameters (i.e., $t_0$, $CFI_{midpoint}$, and $CFI_{last}$; $t_{last}$ is a constant determined by the dataset), the following system of algebraic equation needs to be solved:

$$\begin{cases} 0 = a + bt_0 + ct_0^2 \\ CFI_{mid-point} = a + b(t_0 + \frac{t_{last}-t_0}{2}) + c(t_0 + \frac{t_{last}-t_0}{2})^2 \\ CFI_{last} = a + bt_{last} + ct_{last}^2 \end{cases} \qquad \text{(S2)}$$

The Maple software (https://www.maplesoft.com/) was used to replace a, b, and c in equation S1 as:

$$a = ((t_{last} * CFI_{last} - 4 * CFI_{mid-point} * t_{last} + t_0 * CFI_{last}) * t_0) / (t_{last}\hat{}2 - 2 * t_0 * t_{last} + t_0\hat{}2)$$

$$b = (-(t_{last} * CFI_{last} - 4 * CFI_{mid-point} * t_{last} + 3 * t_0 * CFI_{last} - 4 * CFI_{mid-point} * t_0))/((t_{last} - t_0)^2)$$

$$c = (2 * (-2 * CFI_{mid-point} + CFI_{last}))/(t_{last}\hat{}2 - 2 * t_0 * t_{last} + t_0\hat{}2)$$

Re-parameterizations of other functions of the target CFI are described in detail in the R-code associated to this article (DOI: 10.5281/zenodo.3366107).

**S2. Dealing with missing data**

Missing data are defined as the data during a period for which there are no feed intake records. Missing data may be caused by a power outage, an insensitive sensor, or loss of an identifying ear tag. Ignoring missing data would result in inappropriate CFI data, because the CFI curve would be shifted downward and the missing data could be identified as a perturbation.

To correct for missing data we assumed that there is a continuous pattern of CFI data just before and after the period of missing data. When data are missing for n days, data for n+1 days before and n+1 days after the period of missing data were used to perform a quadratic regression with a model that included a downward shift in CFI associated with the period of missing data. The estimated downward shift in CFI was used to calculate the missing feed intake data. The equation to estimate missing data is written as follow:

$$CFI(t) = \begin{cases} a1 + bt + ct^2, \ t < missing\ day(s) \\ a2 + bt + ct^2, \ t > missing\ day(s) \end{cases} \quad \text{(S3)}$$

Where “t” is the age of the animal (days) and “CFI(t)” is the CFI value at day “t”. Parameters a1 and a2 indicate intercepts of the quadratic function at the days before

and after missing data, respectively. The estimated difference between a1 and a2 is attributed to the cumulative value of daily feed intake during the missing days.

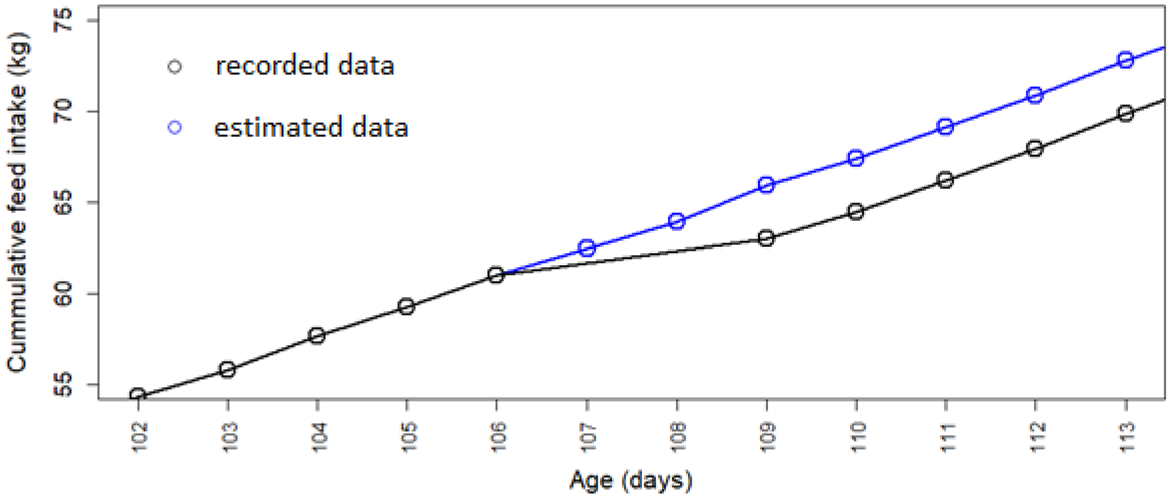


**Figure S1** An example of missing data estimation. Feed intake records for days 107 and 108 were missing and resulted in a downward shift in cumulative feed intake. The missing data were estimated as the downward shift in cumulative feed intake of a quadratic regression between the three days before and the three days after the days associated with missing data.
